# Effect of Cerebral Ischemic Strokes in Different Cerebral Artery Regions on Left Ventricular Function

**DOI:** 10.3389/fcvm.2022.782173

**Published:** 2022-03-08

**Authors:** Li-Juan Zheng, Xin Lin, Yun-Jing Xue

**Affiliations:** ^1^Department of Radiology, Fujian Medical University Union Hospital, The School of Medical Technology and Engineering, Fujian Medical University, Fuzhou, China; ^2^Department of Neonatology, Fujian Maternity and Child Health Hospital, Affiliated Hospital of Fujian Medical University, Fuzhou, China; ^3^Department of Radiology, Fujian Medical University Union Hospital, Fuzhou, China

**Keywords:** cerebral ischemic strokes, cerebral artery regions, left ventricular function, echocardiography, post-stroke cardiovascular complications

## Abstract

**Objectives:**

The relationship between cerebral ischemic stroke and left ventricular function evaluated by echocardiography has been emphasized. Whether lesions in different cerebral artery regions would result in left ventricular dysfunction remains uncertain.

**Methods:**

Patients were divided into middle cerebral artery (MCA) (*n* = 79), posterior cerebral artery (PCA) (*n* = 64), basilar artery (BA) regions (*n* = 66), and no-ischemic stroke group (*n* = 209). We retrospectively collected demographic characteristics, hematologic parameters, and ECG results, and a comparison of echocardiographic parameters was performed to determine the relationship between ischemic stroke and left ventricular function.

**Results:**

A total of 418 patients were included. Demographic characteristics did not significantly differ between the ischemic stroke and non-ischemic stroke groups, except for a history of drinking (*p* < 0.001). Homocysteine levels in the MCA group were higher than those in the PCA and BA groups (*p* < 0.05). The highly sensitive C-reactive protein (hs-CRP) level was higher in the ischemic stroke group than in the non-ischemic stroke one (*p* = 0.001). A higher incidence of ST-T changes in the ECG and lower levels of potassium and magnesium in the ischemic stroke group were found. Significant differences in diastolic function between groups were noted, and the early mitral inflow velocity, annular early diastolic velocity, and ratio between the mitral annular early diastolic velocity and mitral annulus atrial inflow velocity in the MCA group were lower than those in the BA group (*p* < 0.05).

**Conclusions:**

Ischemic strokes exhibited a negative effect on left ventricular diastolic function by echocardiography, especially in MCA region infarcts. These results are of great importance for neurologists as they highlight the need for left ventricular function evaluation after stroke to regulate therapy strategies in time.

## Highlights

- History of drinking, increased levels of homocysteine (HCY) and highly sensitive C-reactive protein (hs-CRP), and ST changes in ECG were more likely occurred in the patients with ischemic stroke.
- Paying more attention to the decreased [Mg^2+^] and [K^+^] levels of patients with strokes.
- Ischemic strokes exhibited a negative effect on the left ventricular diastolic function in the middle cerebral artery (MCA) regions.

## Introduction

Over the past 10 years, the increased incidence of cerebral ischemic strokes has been attributed to behavioral, metabolic, and environmental factors (1). More than 1.5 million people die of post-stroke cardiovascular complications worldwide annually, such as myocardial infarction, unstable angina, congestive heart failure, and coronary artery diseases (2). The changes in the ECG (3) and increased levels of hematologic indicators, such as creatine kinase (CK), creatine kinase isoenzyme (CK-MB), aspartate aminotransferase (AST), and lactate dehydrogenase (LDH) (4) may be used to predict post-stroke cardiovascular complications.

The cardiac complications of strokes are dependent on their locations. Strokes of the insular lobe and basal ganglia, located in the middle cerebral artery (MCA) region, can lead to the loss of cardiac rhythm control, variability to fatal arrhythmias, and death (5, 6). Thalamic strokes, located in the posterior cerebral artery (PCA) region, can cause disturbance of the autonomic nervous system in Takotsubo cardiomyopathy (7). The nucleus ambiguous and nucleus dorsalis, located in the basilar artery (BA) region, convey parasympathetic modulation of the central cardiovascular system (8). In the event of a stroke, the balance between parasympathetic and sympathetic modulation is disrupted, leading to the coronary artery spasm and myocardial ischemia (9).

In recent years, studies have shown the association between stroke and cardiac imaging (10). Echocardiography was selected to guide secondary prevention in the recently updated American Heart Association guidelines on acute stroke management (11). The 90-day mortality after acute ischemic stroke can be predicted by a higher ratio between early mitral inflow and annular early diastolic velocities (E/e'), indicating diastolic dysfunction (12). A lower left ventricular ejection fraction (LVEF), indicative of systolic dysfunction, has been associated with a higher prevalence of silent cerebral infarction (13).

Previous studies have focused on the relationship between different cerebral vascular ischemic regions and myocardial ischemia or arrhythmia through ECG and hematologic indicators without a systematic evaluation of the left ventricular diastolic or systolic function through echocardiography. Moreover, it remains unclear whether infarct lesions in these regions would result in left ventricular dysfunction. Our study aimed to determine the relationship between cerebral ischemic strokes in different arterial regions and left ventricular dysfunction.

## Materials and Methods

### Study Population

This study was conducted in our center (Ethics approval number: 2021KY111), the largest comprehensive stroke treatment hospital in Fujian Province, China, with an annual load of 109,070 outpatients and abundant clinical practice experience. To evaluate post-stroke cardiovascular complications, brain MRI and echocardiography are routinely performed for patients within 24–48 h after stroke.

**Figure 1:**
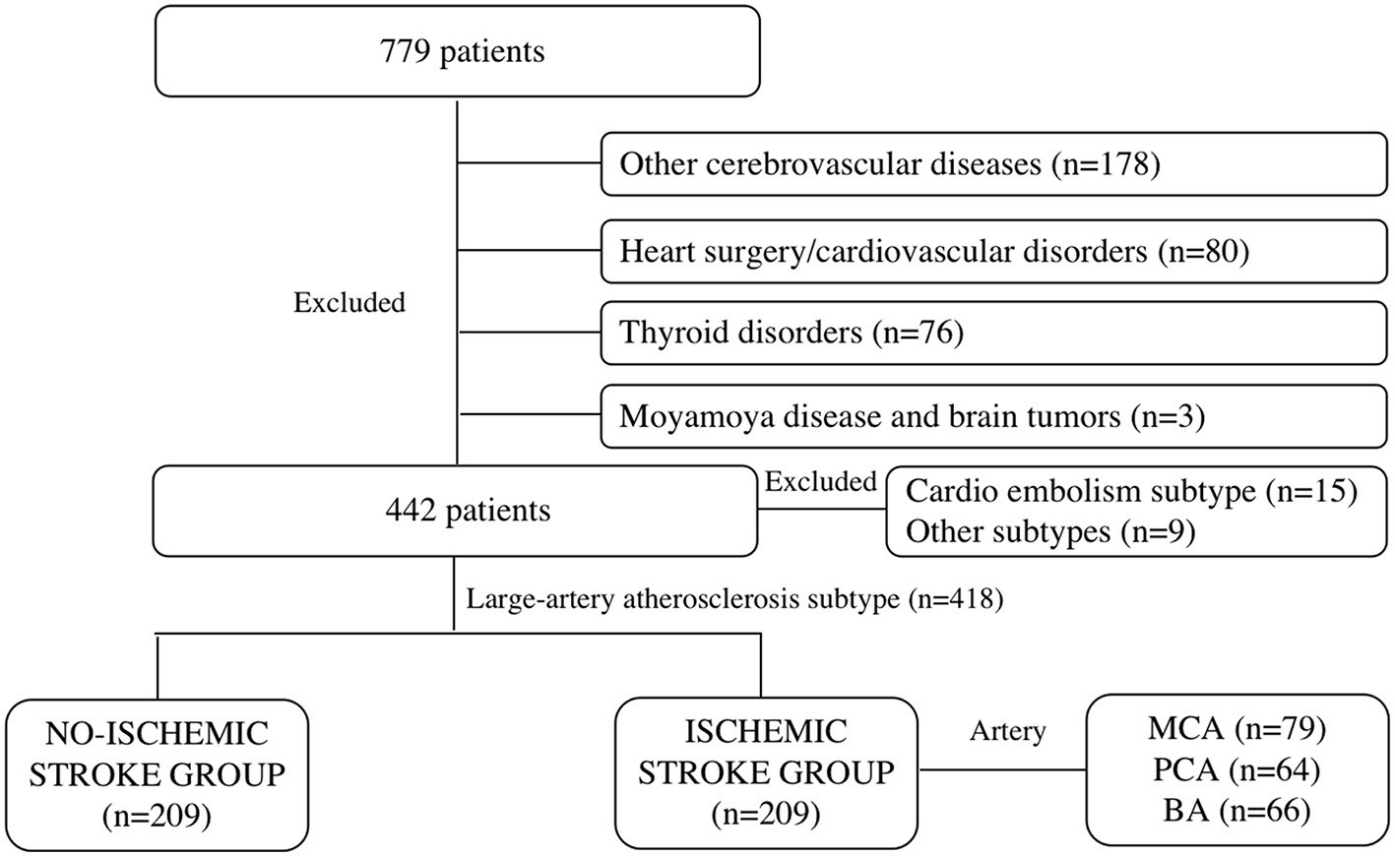
Flow diagram for patient population of ischemic stroke.

Patients with a series of symptoms (limb numbness, blurred vision, dizziness, and nausea) that were admitted to the Department of Neurology of our institution within 24 h from April 1, 2017 to December 31, 2020 were retrospectively included. The exclusion criteria were patients with other cerebrovascular diseases, such as hemorrhagic stroke, multiple regional cerebral ischemic strokes (>1 vascular territory), ruptured cerebral hemangioma, and cerebrovascular malformations. Those who had undergone cardiac surgery (valve replacement, intracoronary stent implantation, coronary artery bypass grafting, and pacemaker implantation) or were diagnosed with cardiovascular disorders, such as congenital heart diseases (ventricular septal defect, cardiomyopathy, atrial septal defect, and patent ductus arteriosus), arrhythmia, myocardial infarction, rheumatic, hypertensive, coronary, and senile valvular heart diseases were excluded. Other exclusion criteria were patients with thyroid disorders (hyperthyroidism, hypothyroidism, hyperparathyroidism, Graves' disease, and thyroid cancer), Moyamoya disease, and brain tumors (glioma, meningioma, ependymoma, germ cell tumor, and metastatic tumor). A total of 779 patients were initially enrolled. After selection, 178 were excluded due to other cerebrovascular diseases, 80 due to cardiac surgery or cardiovascular disorders, 76 due to thyroid disorders, and 3 due to Moyamoya disease and brain tumors (Figure 1).

Primarily included patients underwent brain MRI, ECG, echocardiography, and hematological examinations within 24–48 h after hospitalization, and 231 patients had defined signs of ischemic infarcts in the brain MRI. According to the Trial of Org 10172 in Acute Stroke Treatment (TOAST) Classification of Subtypes of Ischemic Stroke (14), patients with the reasons of large-artery atherosclerosis were included in the ischemic stroke group (*n* = 209), while patients in the parts of cardio embolism subtype (*n* = 15) or other subtypes (*n* = 9) were excluded, through brain MRI examination and echocardiography. Patients were included in the no-ischemic stroke group (*n* = 209) because of no evidence of stoke formation and cardiac function damage were confirmed by brain MRI, echocardiography, or other hematological examinations. What's more, the explanations for the suspicious cerebral ischemic stroke symptoms in the no-ischemic stroke group overwork (*n* = 40), sleep deprived (*n* = 16), having symptoms of limb numbness (*n* = 36), blurred vision (*n* = 32), or dizziness and nausea (*n* = 41) because of temporary increased blood pressure (BP) and cervical spondylopathy without any discoveries of organic diseases (*n* = 44), and all of them received clinical observation and were discharged without any clinical therapy. Patients in the ischemic group received standard medical care according to the European Stroke Organization guidelines diagnosis (15), such as basic treatments and specialty specific treatments (16) for a week or longer. The demographic characteristics of patients were collected, such as sex, age, height, weight, BP grade (17), history of smoking, drinking, and type 2 diabetes.

### Hematologic Parameters and ECG

Patients underwent peripheral venous blood sampling (2–4 ml) when they were hospitalized. Hematologic examination results were recorded and included routine blood parameters (hematocrit and platelet [PLT]), coagulation (prothrombin time and activated partial thromboplastin time), myocardial function (CK-MB, AST, LDH, and alanine aminotransferase), liver function [albumin (ALB), triglycerides, and homocysteine (HCY)], kidney function [blood urea nitrogen, creatinine (Cre)], electrolytes (sodium [Na^+^], calcium [Ca^2+^], potassium [K^+^], and magnesium [Mg^2+^]), and highly sensitive C-reactive protein (hs-CRP). In addition, normal ECG findings and ST-T changes were included. ST-T changes were defined as T wave inversion and ST segment depression (≤0.05 mV).

**Figure 2:**
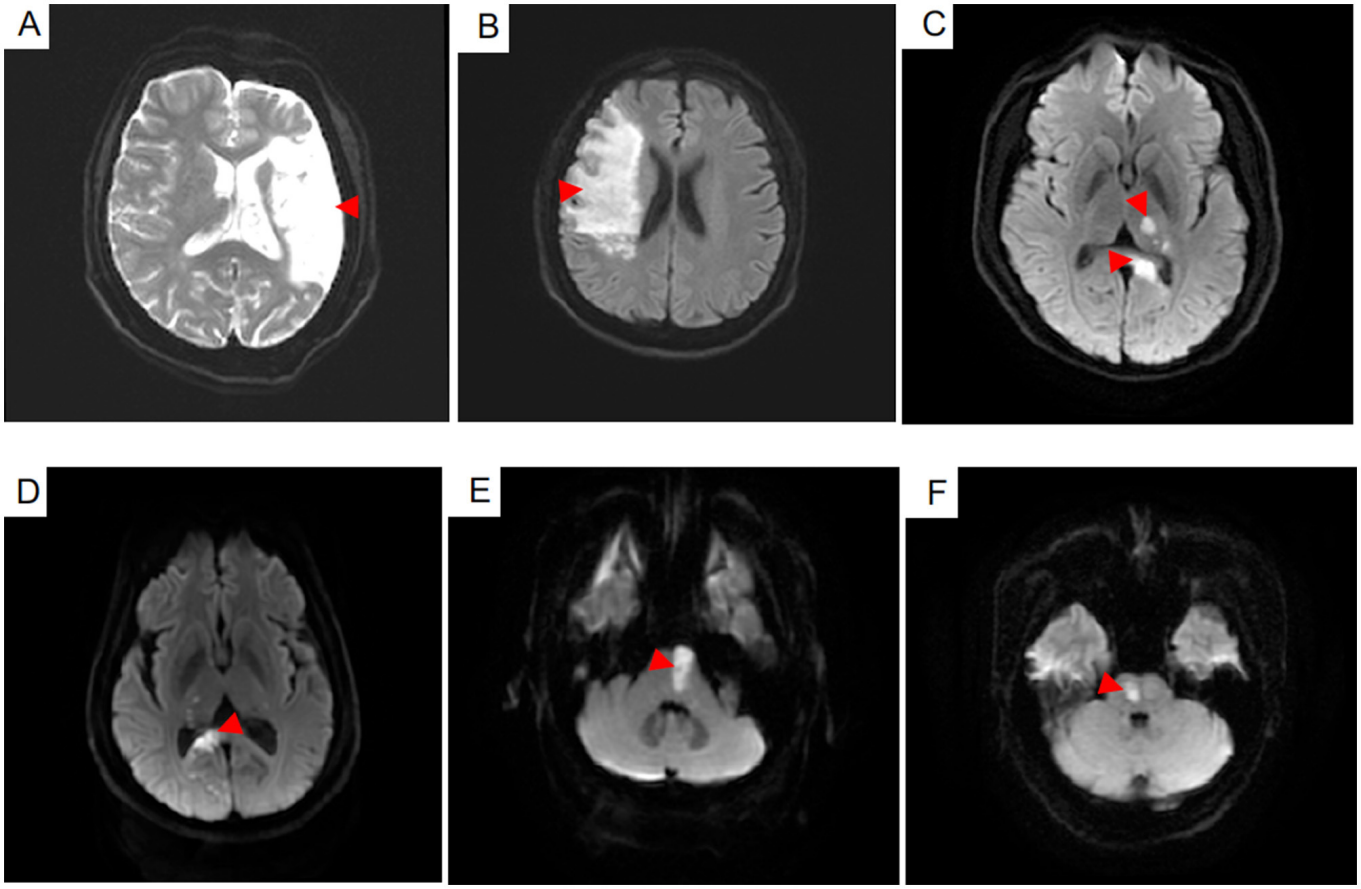
The infarct lesions of six patients in the different cerebral artery regions were shown on the diffusion weighted imaging sequence **(A–F)**. Infarct lesions in the middle cerebral artery the regions including the upper lateral surface of the cerebral hemisphere and the insular lobe were showed in the **(A,B)**. Infarct lesions in the posterior cerebral artery regions including the thalamus, par hippocampal gyrus, inferior temporal gyrus and lingual gyrus were shown in the **(C,D)**. Infarct lesions in the basilar artery regions including the pons and medulla were shown in the **(E,F)**.

**Figure 3:**
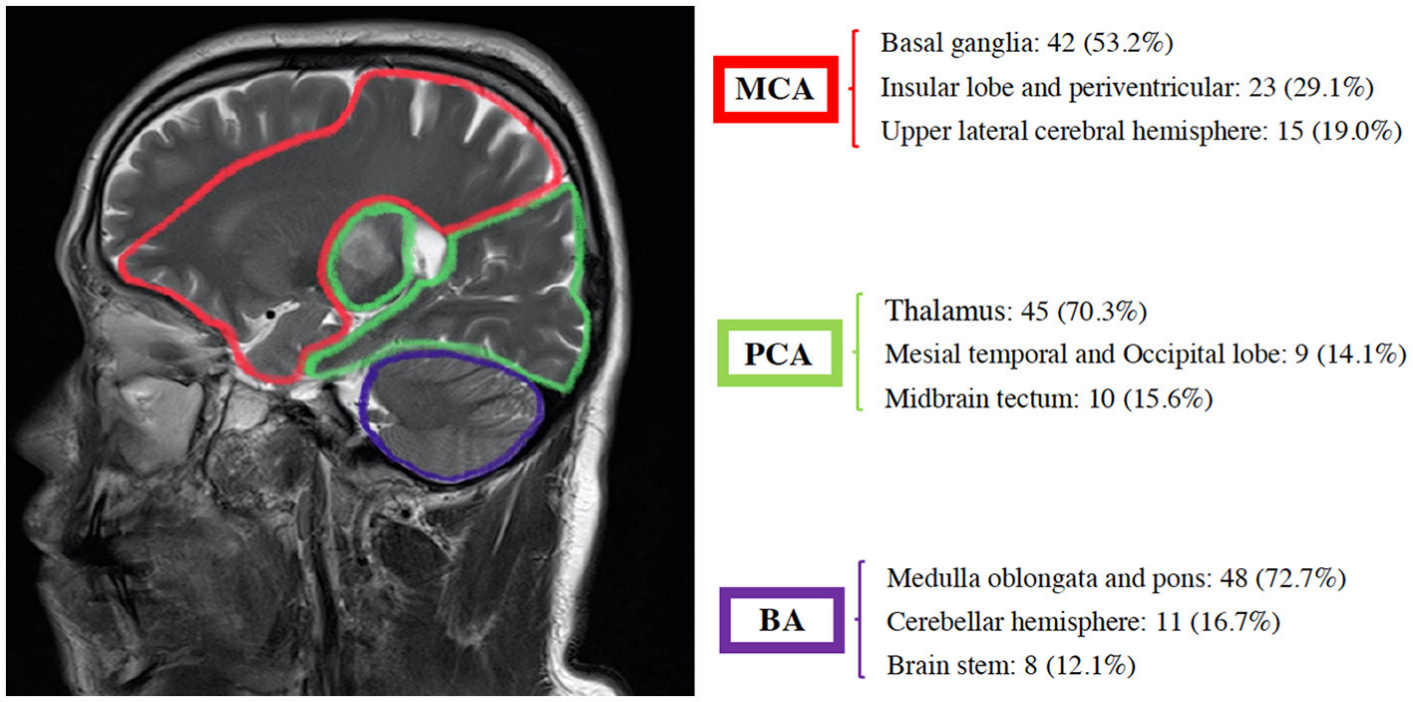
Distribution characteristics of infarct lesions in each cerebral artery region.

Brain imaging was done on a 3.0 T MRI scanner (Discovery 750w, General Electric Healthcare, USA), using a 32-channel head coil for cranial axis T1 weighted, T2 weighted, T2 fluid-attenuated inversion recovery, star-weighted angiography, and diffusion-weighted imaging. Two radiologists with 10 years of experience in imaging diagnosis drew the location and scope of infarction and determined the single ischemic cerebral artery based on the above sequence (Figures 2, 3). Patients with single infarct lesions that were first discovered in the MCA, PCA, or BA were included in the study.

### MRI Diagnosis

Based on the clinical symptoms and MRI findings, patients were divided into ischemic and non-ischemic stroke groups. Furthermore, patients in the former group were categorized into three subgroups (MCA, PCA, and BA) based on the lesion location (Figure 1).

### Echocardiographic Parameters

Echocardiographic parameters were measured by ultrasound physicians with more than 10 years of experience in cardiac diagnosis. Each report was checked and approved by a chief ultrasound physician with more than 20 years of experience before being published. Transthoracic echocardiography was performed using the Voluson E10 machine (General Electric Healthcare ultrasound, Austria), and the chosen Doppler probe frequency was 2.5–3.0 MHz. To measure the diameters of the right ventricle (RV), left atrium (LA), aorta (AO), and pulmonary artery (PA), we chose the standard parasternal long-axis and short-axis views using two-dimensional (2D) windows. We measured the interventricular septal thickness (IVST) and the left ventricular posterior wall thickness at end-diastole (LVPWd) from the parasternal long-axis view at the level of the mitral valve leaflet tips perpendicular to the left ventricular long-axis using 2D windows. The sampling line was perpendicular to the IVS and LVPW and measured the left ventricular internal diameter at end-diastole (LVIDd) and systole (LVIDs) using m-mode windows from the standard parasternal short-axis views. Fractional shortening (FS), indicated that the left ventricular systolic function was calculated using the following formula:


$$\begin{array}{l} \text{FS}\;\left(\text{\%}\right)=\frac{\text{LVIDd}-\text{LVIDs}}{\text{LVIDd}} \end{array}$$

The left ventricular end-diastolic volume (EDV) and end-systolic volume (ESV) were measured using m-mode windows from the standard parasternal short-axis view. LVEF was calculated using the following formula:


$$\begin{array}{l} \text{LVEF}\;\left(\text{\%}\right)=\frac{\text{EDV}-\text{ESV}}{\text{EDV}} \end{array}$$

Where EDV and ESV indicate end-diastolic and end-systolic volumes, respectively. Stroke volume (SV) was calculated as the difference between the EDV and ESV. The heart rate (HR) was defined as the rate of heart beats per minute. Body surface area (BSA) was calculated based on the height and weight using the following formula:


$$\begin{array}{l} \text{BSA}\;\left({\text{m}}^{2}\right)=\left(0.00661\;\times \text{height}\;\left(\text{cm}\right)\right)\;\\ \;+\left(0.012\;\times \text{weight}\;\left(\text{kg}\right)\right)-0.1529 \end{array}$$

Cardiac output (CO) and cardiac index (CI) were measured using the following formulas:


$$\begin{array}{l} \text{CO}\;\left(\frac{\text{L}}{\text{min}}\right)=\text{SV}\times \text{HR}\;\\ \;\text{CI}=\frac{\text{CO}}{\text{BSA}} \end{array}$$

Relative wall thickness (RWT) left ventricular mass (LVM) and LVM index (LVMI) were measured using the adult standard recommended by the American Echocardiography Association (18). To calculate the LVM, we chose the linear method and the following cube formula:


$$\begin{array}{l} \text{LVM}\;\left(\text{g}\right)=0.8\times 1.04\;\\ \;\times ({\left(\text{IVST}+\text{LVIDd}+\;\text{LVPWd}\right)}^{3}-\text{LVID}{\text{d}}^{3})+0.6 \end{array}$$

The LVMI was indexed to BSA and calculated using the following formula:


$$\text{LVMI}\;\left(\frac{\text{g}}{{\text{m}}^{2}}\right)=\frac{\text{LVM}}{\text{BSA}}$$

Left ventricular hypertrophy (LVH) was defined as LVMI > 95 g/m^2^ in women and > 115 g/m^2^ in men (19). Septal hypertrophy (SH) was defined as IVST > 15 mm (20). The Doppler probe was placed at the location of the mitral valve cusp to measure early mitral inflow (E) and late mitral diastolic phase (A) velocities using pulsed wave windows from the standard apical views. The probe was placed at the location of the mitral valve annulus on the sidewall of the ventricle or the septum to measure mitral annular early diastolic (e′) and atrial inflow (a′) velocities using tissue Doppler imaging windows from the standard apical views. Peak flow rate ratio in early and late diastolic phases (E/A), the ratio between mitral annular early diastolic and atrial inflow velocities (e′/a′), and E/e′ were calculated using the above values.

### Statistical Analysis

The statistical analyses were performed using the SPSS Statistic version 26.0 (IBM Corp, Armonk, NY, USA). All demographic characteristics and clinical data were expressed as mean ± SD. In analyses of continuous data, if fit to normal distribution, analyzed by the *t*-test (*n* = 2) or one-way ANOVA test (*n* > 2); and if not, analyzed by Wilcoxon test (*n* = 2) or Kruskal–Wallis *H*-test (*n* > 2). The differences of echocardiographic parameters among different cerebral artery groups were shown by box diagrams. Chi-square tests or Fisher's exact tests were given in the analyses of categorical variables as appropriate. Results with *p* < 0.05 were considered statistically significant.

## Results

A total of 418 patients were included in the study, 209 of whom were in the ischemic stroke group based on the brain MRI and clinical diagnosis. These patients were further subdivided into MCA (*n* = 79), the PCA (*n* = 64), and BA (*n* = 66) groups based on the location of the infarct (Figures 2, 3).

There was no significant difference in the sex between patients in the ischemic stroke and non-ischemic stroke groups (*p* = 0.557). The average ages of each group were not significantly different (*p* = 0.132) and were (64 ± 11) years in the non-ischemic stroke, (64 ± 12) years in the MCA, (62 ± 11) years in the PCA, and (60 ± 13) years in the BA groups. As shown in Table 1, the height and weight of each group of patients were similar (*p* = 0.147 and *p* = 0.051, respectively), as was the BA group (*p* = 0.051). Patients in the ischemic stroke group were significantly more likely to have a history of drinking compared with those in the non-ischemic stroke group (*p* < 0.001). No differences were found in terms of the history of smoking and type 2 diabetes between the two groups (*p* = 0.179 and *p* = 0.559, respectively).

**Table 1:** The demographic characteristics, hematologic parameters and electrocardiogram between the ischemic stroke group (MCA, PCA, BA) and the no-ischemic stroke group.

**Variables**	**No-ischemic stroke**	**MCA**	**PCA**	**BA**	***P***
	**(*n* = 209)**	**(*n* = 79)**	**(*n* = 64)**	**(*n* = 66)**	
**Demographic characteristics**
Male/Female	113 (54.1)	42 (53.2)	31 (48.4)	40 (60.6)	0.577
Age (year)	64 ± 11	64 ± 12	62 ± 11	60 ± 13	0.132
Height (cm)	163.7 ± 7.0	162.7 ± 8.1	161.5 ± 8.9	164.4 ± 6.8	0.147
Weight (kg)	61.8 ± 10.1	64.1 ± 10.1	62.9 ± 10.1	65.4 ± 9.8	0.051
Normal blood pressure	56 (26.8)	16 (20.3)	11 (17.2)	13 (19.7)	0.145
Elevated blood pressure	55 (26.3)	15 (19.0)	12 (18.8)	10 (15.2)	
Hypertension stage I	47 (22.5)	21 (26.6)	15 (23.4)	19 (28.8)	
Hypertension stage II	51 (24.4)	27 (34.2)	26 (40.6)	24 (36.4)	
Smoking (Y/N)	48 (23.0)	23 (29.1)	22 (34.4)	22 (33.3)	0.179
Drinking (Y/N)	10 (4.8)	11 (13.9)	13 (20.3)	10 (15.2)	<0.001
Type 2 diabetes (Y/N)	44 (21.1)	13 (16.5)	12 (18.8)	17 (25.8)	0.559
**Hematologic parameters and electrocardiogram (ECG)**
HCT (%)	40.1 ± 3.8	41.0 ± 3.7	40.6 ± 4.1	41.4 ± 3.8	0.138
PLT (10∧9/L)	222 ± 45	223 ± 41	219 ± 42	215 ± 41	0.939
PT (sec)	12.5 ± 0.6	12.5 ± 0.6	12.5 ± 0.6	12.5 ± 0.7	0.889
APTT (sec)	34.74 ± 3.04	34.47 ± 2.97	34.49 ± 3.45	34.74 ± 2.89	0.872
D-dimer (mg/L)	0.34 ± 0.19	0.40 ± 0.25	0.36 ± 0.18	0.40 ± 0.26	0.086
CKMB (U/L)	14.1 ± 4.1	14.4 ± 5.2	14.5 ± 4.1	15.4 ± 5.4	0.227
ALT (U/L)	19.5 ± 7.9	19.5 ± 7.9	19.1 ± 7.6	19.1 ± 8.0	0.911
AST (U/L)	20.7 ± 6.0	21.5 ± 5.9	20.2 ± 6.1	20.1 ± 7.0	0.296
ALB (g/L)	40.7 ± 3.5	40.2 ± 3.3	39.9 ± 3.3	40.2 ± 3.3	0.093
BUN (mmol/L)	5.0 ± 1.1	4.8 ± 1.1	4.7 ± 1.1	4.7 ± 1.1	0.172
Cre (μmoI/L)	70.5 ± 15.1	71.6 ± 15.1	68.1 ± 16.4	70.1 ± 16.9	0.177
TG (mmol/L)	1.16 ± 0.36	1.23 ± 0.32	1.21 ± 0.32	1.17 ± 0.29	0.420
HCY (μmol/L)	8.35 ± 1.93	8.89 ± 2.32	7.99 ± 2.20	8.09 ± 2.54	0.040
hs-CRP (mg/L)	1.72 ± 1.67	2.66 ± 2.22	2.47 ± 1.92	1.76 ± 1.54	0.001
LDH (U/L)	170 ± 30	179 ± 33	177 ± 35	174 ± 36	0.211
Na^+^ (mmol/l)	141.2 ± 3.3	140.7 ± 2.4	141.1 ± 2.6	141.0 ± 7.2	0.177
Ca^2+^ (mmol/l)	2.29 ± 0.11	2.29 ± 0.12	2.28 ± 0.11	2.29 ± 0.11	0.911
K^+^ (mmol/l)	4.06 ± 0.34	3.98 ± 0.27	4.01 ± 0.35	3.93 ± 0.32	0.021
Mg^2+^ (mmol/l)	0.88 ± 0.06	0.87 ± 0.08	0.86 ± 0.07	0.86 ± 0.07	0.037
ST-T changes in ECG (Y/N)	24 (11.5)	29 (36.7)	12 (18.8)	22 (33.3)	<0.001

*MCA, middle cerebral artery; PCA, posterior cerebral artery; BA, basilar artery; HCT, hematocrit; PLT, platelet; PT, prothrombin time; APTT, activated partial thromboplastin time; CKMB, creatine kinase isoenzyme; ALT, alanine aminotransferase; AST, aspartate aminotransferase; ALB, albumin; BUN, blood urea nitrogen; Cre, creatinine; TG, triglycerides; HCY, homocysteine; hs-CRP, high sensitive C-reaction; LDH, lactate dehydrogenase; Na^+^, natrium; Ca^2+^, calcium; K^+^, kalium; Mg^2+^, magnesium; Y, yes; N, no*.

As shown in Table 1, there were no significant differences in the routine blood, coagulation, myocardial function, and kidney function parameters between the two groups (*p* > 0.05). In the analysis of liver function parameters, only HCY of the MCA group (8.89 ± 2.32 μmol/L) was significantly higher than that of the PCA (7.99 ± 2.20 μmol/L, *p* = 0.016) and BA groups (8.09 ± 2.54 μmol/L, *p* = 0.013). In comparison with the non-ischemic stroke group, the levels of [K^+^] and [Mg^2+^] were significantly lower in the ischemic stroke group (*p* = 0.021 and *p* = 0.037, respectively), but no difference was noted in the levels of [Na^+^] and [Ca^2+^] (*p* = 0.177 and *p* = 0.991, respectively). Hs-CRP levels were higher in the ischemic than in the non-ischemic stroke group (*p* = 0.001), especially in the MCA (2.66 ± 2.22 mg/L) and PCA groups (2.47 ± 1.92 mg/L). The incidence of ST-T changes on ECG was also higher in the ischemic stroke group (*p* < 0.001).

Lesions located in the basal ganglia, insular lobe, periventricular, and upper lateral cerebral hemispheres were included in the MCA group. Those located in the thalamus, mesial temporal, occipital lobe, and midbrain tectum were included in the PCA group. Finally, those located in the medulla oblongata, pons, cerebellar hemisphere, and brain stem were included in the BA group. The majority of stroke incidence regions were the basal ganglia in the MCA (53.2%), thalamus in the PCA (70.3%), and medulla oblongata and pons in BA (72.7%) as shown in Figure 3.

**Figure 4:**
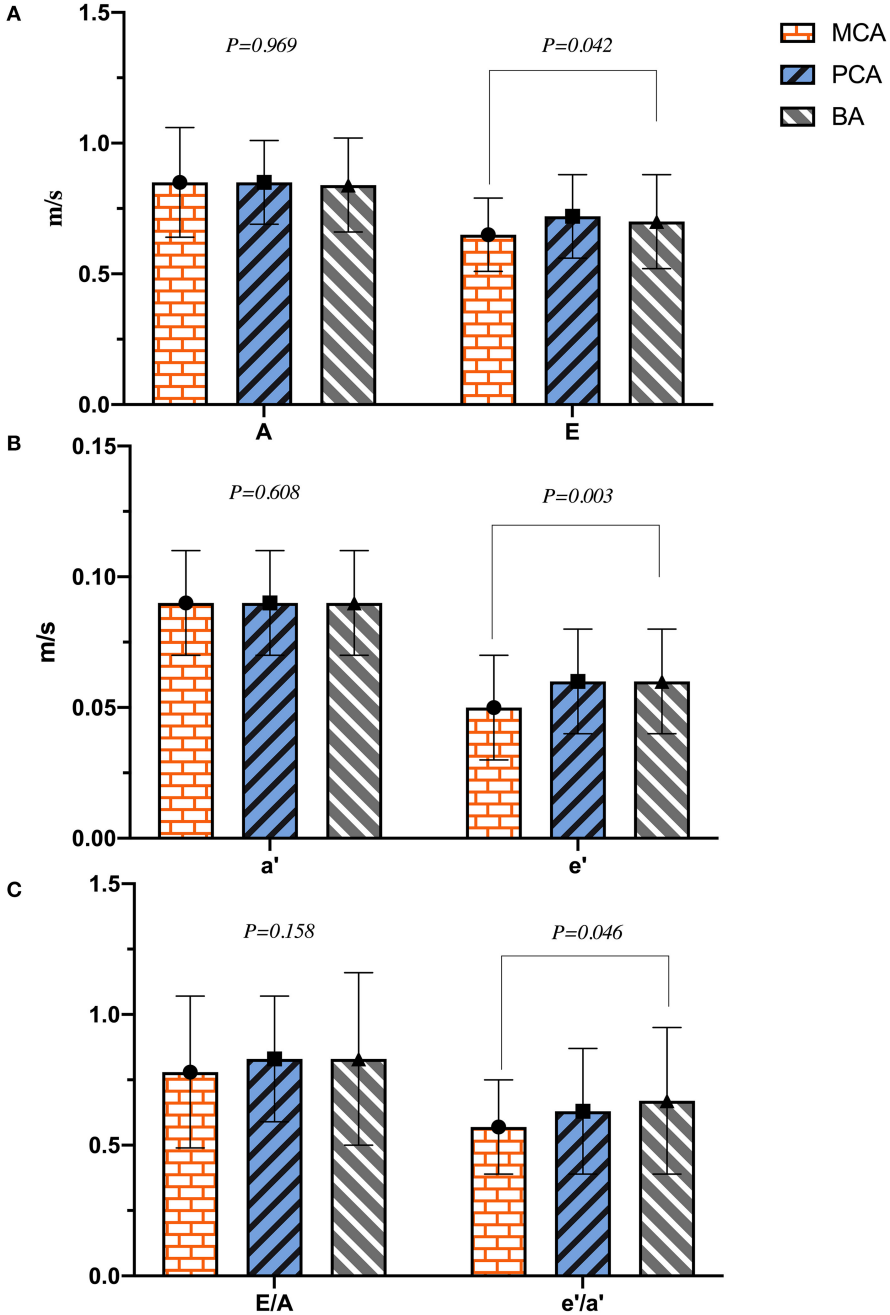
The plot of **(A–C)** indicated the difference of left ventricular diastolic function in different cerebral artery groups.

**Figure 5:**
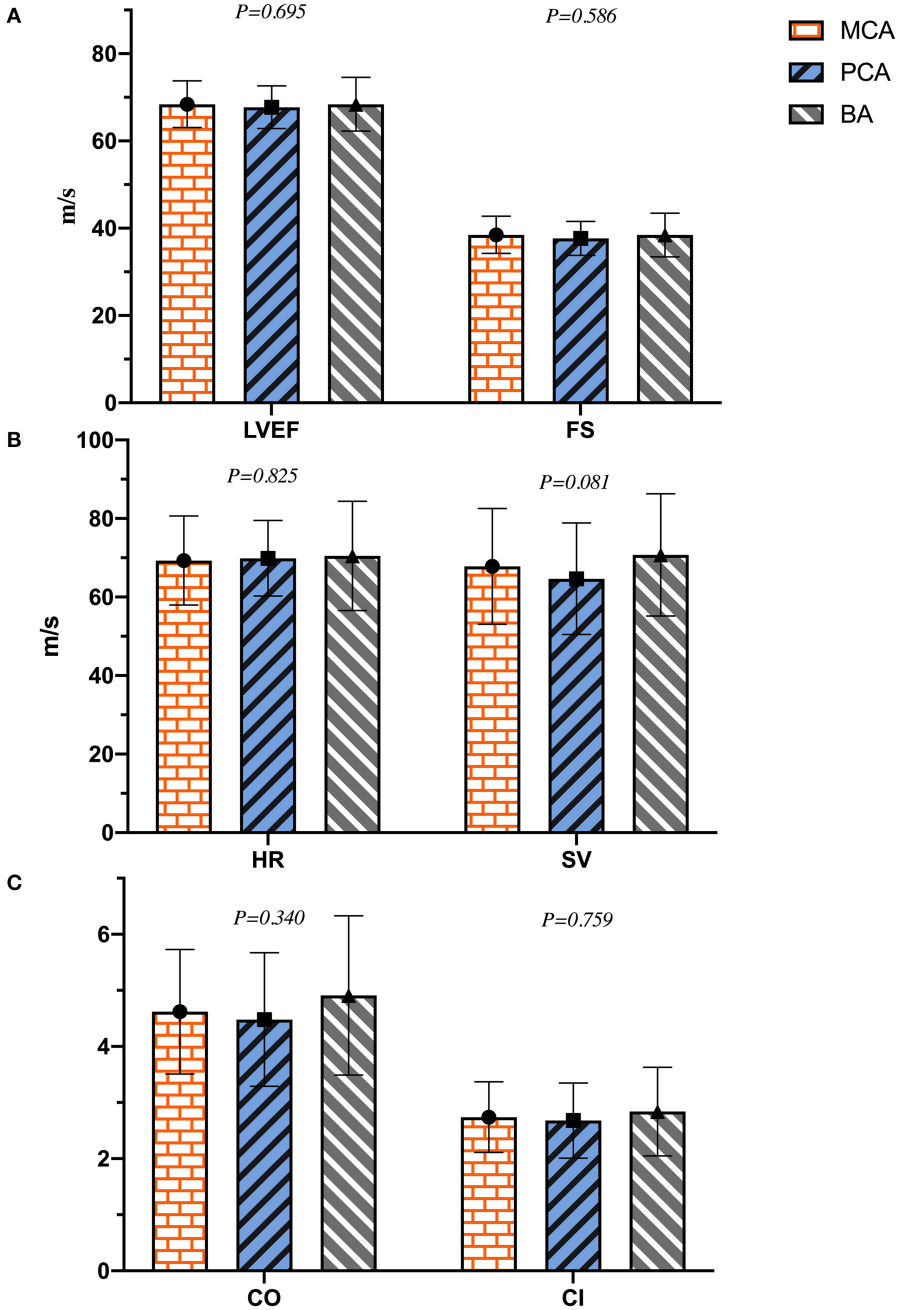
The plot of **(A–C)** indicated the difference of left ventricular systolic function in different cerebral artery groups.

As shown in Table 2, the diameters of RV, LA, AO, PA, IVST, LVPWd, and LVIDd were significantly different between the two groups (*p* < 0.01), and LVIDd in the PCA group was much smaller than that of the BA group (45.4 ± 4.2 vs. 47.0 ± 3.9 mm, *p* = 0.023). LVM, LVMI, and RWT in the ischemic stroke group were significantly higher than in the non-ischemic one (*p* < 0.001). No differences were found in LVIDs between the two groups (*p* = 0.220). Patients with ischemic stroke had a lower HR than those without (*p* = 0.043). Except for HR, there were no significant differences in left ventricular systolic function (LVEF, FS, SV, CO, and CI) between the two groups (*p* > 0.05). In the analyses of left ventricular diastolic function, E and E/A in the ischemic stroke group were lower than that of the non-ischemic one (*p* < 0.05), without a significant difference in A (*p* = 0.419). In addition, lower e′, a′, and e′/a′ of the left ventricular motor function were found in the ischemic stroke group (*p* < 0.05). Conversely, E/e′ in the ischemic stroke group was higher than the non-ischemic one (*p* < 0.001). In the comparison of different cerebral arteries, we found that E, e′, and e′/a′ in the MCA group were significantly lower than those in the BA group (*p* < 0.05), as illustrated in Figure 4. No differences of left ventricular systolic function (LVEF, FS, HR, SV, CO, and CI) among MCA, PA, and BA groups are shown in Figure 5. The incidence of LVH, SH, and diastolic dysfunction in the ischemic stroke group was much higher than the non-ischemic one (*p* < 0.05).

**Table 2:** The outcomes of echocardiogram between the ischemic stroke group (MCA, PCA, BA) and no-ischemic stroke group.

**Variables**	**No-ischemic stroke**	**MCA**	**PCA**	**BA**	***P***
	**(*n* = 209)**	**(*n* = 79)**	**(*n* = 64)**	**(*n* = 66)**	
Right ventricle (mm)	19.6 ± 2.0	20.2 ± 2.3	20.2 ± 1.4	20.7 ± 1.6	<0.001
Left atrium (mm)	31.5 ± 4.3	34.2 ± 5.4	33.4 ± 4.4	33.6 ± 4.3	<0.001
Aorta (mm)	28.8 ± 3.4	30.4 ± 3.9	29.8 ± 3.7	30.7 ± 4.0	<0.001
Pulmonary artery (mm)	19.6 ± 1.9	20.6 ± 2.2	20.1 ± 1.5	20.4 ± 1.6	0.002
Interventricular septal thickness (mm)	9.8 ± 1.4	11.1 ± 1.6	10.9 ± 1.7	11.0 ± 1.8	<0.001
LVPWd (mm)	9.4 ± 1.3	10.3 ± 1.3	10.3 ± 1.5	10.3 ± 1.5	<0.001
LVIDd (mm)	45.4 ± 4.1	46.2 ± 4.6	45.4 ± 4.2	47.0 ± 3.9	0.040
LVIDs (mm)	28.0 ± 3.0	28.4 ± 3.9	28.1 ± 2.9	28.8 ± 3.0	0.220
Left ventricular mass (g)	148.8 ± 39.0	177.9 ± 45.4	170.7 ± 44.6	182.7 ± 50.6	<0.001
Left ventricular mass index (g/m^2^)	90.5 ± 20.4	106.7 ± 23.6	103.4 ± 23.9	107.2 ± 27.7	<0.001
Relative wall thickness	0.43 ± 0.07	0.46 ± 0.07	0.46 ± 0.07	0.45 ± 0.07	<0.001
**Left ventricular systolic function**
Left ventricular ejection fraction (%)	68.7 ± 5.1	68.6 ± 5.4	67.7 ± 4.9	68.4 ± 6.1	0.647
Fractional shortening (%)	38.5 ± 4.2	38.5 ± 4.3	37.7 ± 3.9	38.5 ± 5.0	0.595
Heart rate (bpm)	73 ± 11	69 ± 11	70 ± 10	71 ± 14	0.043
Stroke volume (ml)	65.6 ± 14.4	67.8 ± 14.7	64.7 ± 14.2	70.7 ± 15.6	0.050
Cardiac output (L/min)	4.6 ± 1.1	4.6 ± 1.1	4.5 ± 1.2	4.9 ± 1.4	0.507
Cardiac index	2.8 ± 0.6	2.7 ± 0.6	2.7 ± 0.7	2.8 ± 0.8	0.743
**Left ventricular diastolic function**
E (m/s)	0.72 ± 0.18	0.65 ± 0.14	0.72 ± 0.16	0.70 ± 0.18	0.029
A (m/s)	0.82 ± 0.18	0.85 ± 0.21	0.85 ± 0.16	0.84 ± 0.18	0.419
E/A	0.86 ± 0.25	0.78 ± 0.29	0.83 ± 0.24	0.83 ± 0.33	0.015
**Ventricular motor function**
e′ (m/s)	0.07 ± 0.02	0.05 ± 0.02	0.06 ± 0.02	0.06 ± 0.02	<0.001
a′ (m/s)	0.10 ± 0.02	0.09 ± 0.02	0.09 ± 0.02	0.09 ± 0.02	0.010
e′/a′	0.72 ± 0.29	0.57 ± 0.18	0.63 ± 0.24	0.67 ± 0.28	<0.001
E/e′	10.92 ± 3.01	13.66 ± 5.09	13.10 ± 3.48	12.18 ± 3.19	<0.001
**Diagnosis results**
Left ventricular hypertrophy (Y/N)	22 (10.5)	33 (41.8)	25 (39.1)	27 (40.9)	<0.001
Septal hypertrophy (Y/N)	20 (9.6)	19 (24.1)	13 (20.3)	14 (21.2)	0.006
Diastolic dysfunction (Y/N)	38 (18.2)	54 (68.4)	35 (54.7)	38 (57.6)	<0.001

*MCA, middle cerebral artery; PCA, posterior cerebral artery; BA, basilar artery; LVPWd, left ventricular posterior wall thickness at end-diastole; LVIDd, left ventricular internal diameter at end-diastole; LVIDs, left ventricular internal diameter at end-systole; E, early mitral inflow velocity; A, late mitral diastolic phases velocity; E/A, peak flow rate ratio in early and late diastolic phases; e′, mitral annular early diastolic velocity; a′, mitral annulus atrial inflow velocity; e′/a′, ratio between mitral annular early diastolic velocity and mitral annulus atrial inflow velocity; E/e′, ratio between early mitral inflow velocity and mitral annular early diastolic velocity; Y, yes; N, no*.

## Discussion

Imbibing alcohol more than three drinks a day on average increases the risk of ischemic stroke, with a higher risk of mortality than morbidity (21). We found that 49.4% of patients with ischemic stroke had a history of drinking alcohol compared with only 4.8% in the non-ischemic stroke group. Moreover, each patient with a history of drinking alcohol in the ischemic stroke had more than 5 drinks every day for 10 years or more. Heavy drinking alcohol was associated with impaired fibrinolysis, increased platelet activation, and increases in blood pressure and heart rate and these factors increased the incidence of ischemic stroke (22). HCY, a metabolite of methionine formed by the action of invertase, can damage vascular endothelial cells, strengthen the platelet function, cause thrombosis, and increase the risk of cardiovascular diseases and death (23). Higher levels of HCY were noted in the MCA compared with the BA group, which might suggest a higher incidence of post-stroke cardiovascular diseases in the MCA group due to a hypercoagulable state. We also found a higher PLT count in the MCA compared with the BA group. Moreover, hs-CRP levels in the ischemic stroke group were significantly higher, and increased levels have been previously shown to be associated with ischemic stroke (24). Our results showed the decreased levels of [K^+^] and [Mg^2+^] and increased incidence of ST-T changes on the ECG in the ischemic stroke group. These can be explained by the increased levels of cortisol, plasma endothelin, and glucagon, which lead to internal environment imbalance and lower plasma [Mg^2+^] levels, contributing to a higher risk of ischemic stroke (25). It has been suggested that increasing magnesium intake reduces the risk of stroke (26). In addition, post-stroke patients often have poor diets, difficulty swallowing, and acid-base balance disorders, which can lead to abnormal potassium levels in myocardial cells and disrupt cardiac electrical activity. The presence of isolated non-specific ST-segment and T-wave abnormalities in patients with an otherwise normal ECG has been associated with a 27% increased risk of future ischemic stroke (27).

Cardiac activity is innervated and modified by sympathetic and parasympathetic nerves from the hypothalamic-pituitary-adrenal (HPA) axis simultaneously when resting during physical, emotional, and psychological stress (28). If the HPA regions are damaged from an ischemic stroke, it could have a negative effect on the cardiac electrical activity caused by an imbalance in the autonomic nervous system. We found that the HR of patients in the ischemic stroke group was slower than that of the non-ischemic one, which could be related to the parasympathetic nerve dysfunction and its role in damaging the cardiac conduction system, slowing down the HR (29). The worse left ventricular diastolic function, LVH, and SH were found in the ischemic stroke group. In the event of an acute ischemic stroke, a strong stress response in the human body would result in the release of catecholamines, which in turn promote the sympathetic and minimize the parasympathetic activities to intensify constriction of the heart vasculature, coronary artery spasm, and cause cardiac hypertrophy or myocardial ischemia (30).

Long-term increased levels of catecholamines can lead to cardiotoxicity (31) and may generate transient fibrosis and left ventricular diastolic dysfunction (32). In the event of an ischemic stroke involving the hypothalamus, the syndrome of inappropriate antidiuretic hormone secretion occurs and causes water and sodium retention, which translates to an increase in circulating blood volume and cardiac load (33). In addition, we found decreased levels of e′, a′, and e′/a′ in patients with ischemic strokes, indicating left ventricular motor dysfunction. E/e′ indicates a robust surrogate marker of increased left ventricular filling pressure (34) and is predictive of outflow tract obstruction in hypertrophic cardiomyopathy (35). It was found to be higher in patients in the ischemic stroke group compared with the non-ischemic one in our study.

**Figure 6:**
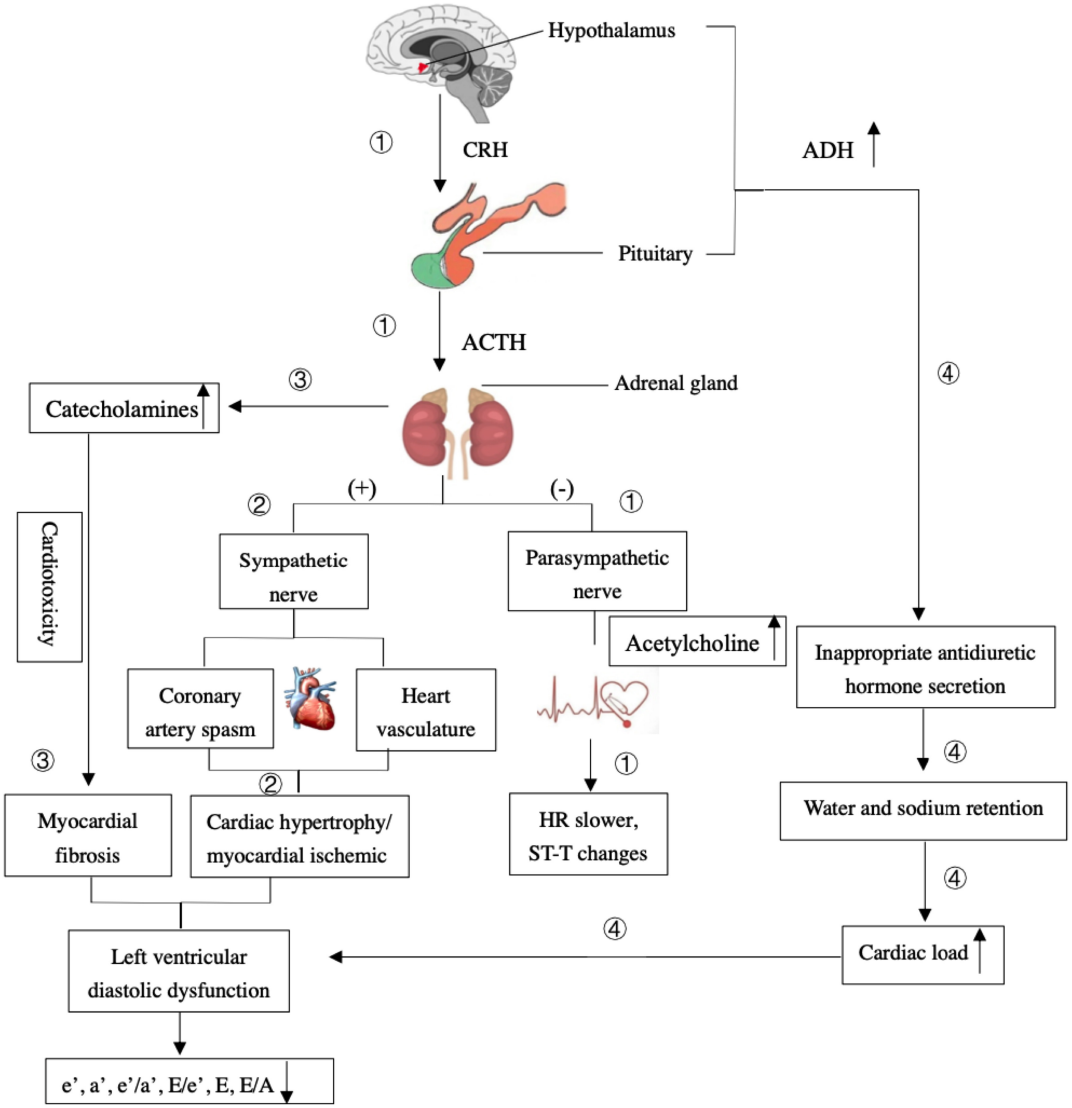
The mechanism of the effects of ischemic stroke on the left ventricular diastolic dysfunction. ① The HPA regions are damaged from an ischemic stroke, it could stimulate the parasympathetic nerve to have a negative effect on the cardiac electrical activity and result in the heart rate (HR) slower and ST-T changes. ② A strong stress response would result in the release of catecholamines, which in turn promote sympathetic and minimize parasympathetic activities to intensify constriction of the heart vasculature, coronary artery spasm, and cause cardiac hypertrophy or myocardial ischemia. ③ Long-term elevated levels of catecholamines can lead to cardiotoxicity and generate transient fibrosis and left ventricular diastolic dysfunction. ④ The excessive release of antidiuretic hormone (ADH) caused by damaged hypothalamus and the syndrome of inappropriate antidiuretic hormone secretion occurs. It causes water and sodium retention, which translates to an increase in cardiac load and left ventricular diastolic dysfunction.

The anterior cerebral artery (ACA), MCA, PCA, and BA are part of the circle of Willis, which is the most important collateral circulatory system in the brain. When the blood in one of these arteries is reduced or blocked, it is redistributed through circular regulation to maintain the blood supply of various parts of the brain. The incidence of ischemic stroke is related to the infarct region of the insufficiently perfused cerebral circulatory system supply (36). We found that the levels of E, e′, and e′/a′ of the MCA group were much lower than those of the BA group in our study, indicating that the ischemic stroke had worse effects on left ventricular diastolic and motor function in the MCA regions. Decreased baroreflex sensitivity and mirroring sympathetic activation might lead to above outcomes. And basal ganglia involvement might be associated with the development of a malignant course of life-threatening edema in MCA infarcts (37). With the mirroring sympathetic activity, the HPA axis stimulates the adrenal glands to release large quantities of catecholamines, resulting in the cardiotoxicity, which is the most important mechanism of post-stroke cardiovascular complications (30). Moreover, the insular cortex located in the MCA region is the main control structure of the heart's autonomic rhythm. Thus, ischemic strokes can lead to fatal arrhythmias and myocardial ischemia (5). Unlike the sympathetic modulation of the central cardiovascular system, which plays a major role in ventricular myocytes, the parasympathetic nerves mainly act on atrial myocytes instead of ventricular ones by releasing acetylcholine in mammals (38). Therefore, when cerebral ischemic strokes occurred, the MCA regions, dominated by sympathetic nerves, had a stronger negative effect on left ventricular function than the BA regions that are dominated by parasympathetic ones. Not only the cerebral ischemic strokes in different cerebral artery regions had a significant impact on left ventricular function (Figure 6), a vice-versa relation was but also found in the choric and acute setting. The mechanism of it in acute setting probably was parasympathetic vasodilatory function, and which was to preserve the cerebral blood flow, decrease ischemic core, promote hemodynamic hypoperfusion, and finally ischemic core extension and stroke recurrence (39, 40). Moreover, the release of plasticity promoting neuromodulators, such as acetylcholine and norepinephrine were stimulated by the vagus nerve triggers, throughout the cortex, and which could be paired with rehabilitation to enhance plasticity and support recovery of upper limb function after chronic stroke (41). Vagus nerve stimulation therapy was promoted and shown to reduce the stroke size in animal models attenuating stroke volumes and post-stroke outcomes studied in rehabilitation only in anterior circulation strokes (42).

The healthy people were seldomly asked for the routine physical examinations, such as brain MRI, ECG, and echocardiography, and only when they had a series of ischemic-stroke-like symptoms, these examinations were done to determine the exact pathogenesis. After comprehensive evaluation, they were not found of any ischemic regions or cardiogenic diseases except for overwork, sleep deprived, having symptoms of limb numbness, blurred vision, or dizziness and nausea because of temporary increased blood pressure and cervical spondylopathy. It was reasonable to compare included patients of both groups because they were having similar demographic characteristics and no cardiovascular diseases before stroke occurred. The occasional overwork and sleep deprived might make the effects on changes in the transient rhythm of the heart other than ventricular function (43). Though the severe increased blood pressure might increase the risk of cerebral hemorrhage and then damage the blood supply of the cardiac muscle, the slight increase of short-term blood pressure accompanied with no cerebral hemorrhage signs had little effect on the ventricular diastolic function. Any negative effects on the ventricular function were not found in the patients with cervical spondylopathy.

Although this was the first study to put forward the effects of stroke in different cerebral artery regions on left ventricular function, because of the lacking in the adequate number of cases and poor prognosis, patients with infract lesions located in the ACA region were not included in the study. Limited on the examinations, the thickness of cardiac ventricular was measured by transthoracic electrocardiography rather than transesophageal electrocardiography in our study, which might have some errors in measurement. Additionally, the long-term follow-up of post-stroke cardiovascular complications is necessary, and we will track it continuously.

History of drinking, increased levels of HCY and hs-CRP, and ST changes in ECG were more likely occurred in the ischemic stroke patients, and we should pay more attention to the decreased [Mg^2+^] and [K^+^] levels of patients with strokes. Ischemic strokes exhibited a negative effect on the left ventricular diastolic function by echocardiography, especially in the MCA region infarcts. These results are of great importance for neurologists as they highlight the need for left ventricular function evaluation after stroke to regulate therapy strategies in time.

## Data Availability Statement

The raw data supporting the conclusions of this article will be made available by the authors, without undue reservation.

## Ethics Statement

The study was approved by the Ethics Committee of Fujian Medical University Union Hospital (Ethics Approval Number: 2021KY111). Written informed consent for participation was not required for this study in accordance with the national legislation and the institutional requirements.

## Author Contributions

L-JZ collected the clinical data and prepared Figures 1–3. XL proposed the idea of this work, prepared Tables 1, 2 and Figures 4–6, and wrote the main manuscript text. Y-JX amended the manuscript text. All authors reviewed the manuscript. All authors contributed to the article and approved the submitted version.

## Funding

This study was supported in part by the Fujian Provincial Science and Technology Innovation Joint Fund Project (2018Y9025), China.

## Conflict of Interest

The authors declare that the research was conducted in the absence of any commercial or financial relationships that could be construed as a potential conflict of interest.

## Publisher's Note

All claims expressed in this article are solely those of the authors and do not necessarily represent those of their affiliated organizations, or those of the publisher, the editors and the reviewers. Any product that may be evaluated in this article, or claim that may be made by its manufacturer, is not guaranteed or endorsed by the publisher.
